# Double Percolation of Poly(lactic acid)/Low-Density Polyethylene/Carbon Nanotube (PLA/LDPE/CNT) Composites for Force-Sensor Application: Impact of Preferential Localization and Mixing Sequence

**DOI:** 10.3390/polym16131906

**Published:** 2024-07-03

**Authors:** Todsapol Kajornprai, Rapisa Jarapanyacheep, Jatupat Saikaeo, Soodkhet Pojprapai, Kasama Jarukumjorn, Tatiya Trongsatitkul

**Affiliations:** 1 Research Center for Biocomposite Materials for Medical Industry and Agricultural and Food Industry, Suranaree University of Technology, Nakhon Ratchasima 30000, Thailand; kajornprai.t@gmail.com (T.K.); rapisa.j@gmail.com (R.J.); jatupat_s@hotmail.com (J.S.); kasama@sut.ac.th (K.J.); 2 School of Ceramic Engineering, Institute of Engineering, Suranaree University of Technology, Nakhon Ratchasima 30000, Thailand; soodkhet@g.sut.ac.th; 3 School of Polymer Engineering, Institute of Engineering, Suranaree University of Technology, Nakhon Ratchasima 30000, Thailand

**Keywords:** carbon nanotube, double percolation, electrical conductivity, selective localization, force sensor

## Abstract

This study explores the enhancement of electrical conductivity in polymer composites by incorporating carbon nanotubes (CNTs) into a co-continuous poly(lactic acid)/low-density polyethylene (PLA/LDPE) blend, creating a double percolation structure. Theoretical thermodynamic predictions indicate that CNTs preferentially localize in the LDPE phase. The percolation threshold of CNTs in the PLA/LDPE/CNT composites was 0.208 vol% (5.56 wt%), an 80% reduction compared to the LDPE/CNT composite, due to the double percolation structure. This thermodynamic migration of CNTs from PLA to LDPE significantly enhanced conductivity, achieving a 13.8-fold increase at a 7.5 wt% CNT loading compared to the LDPE/CNT composite. The localization of CNTs was driven by thermodynamic, kinetic, and rheological factors, with viscosity differences between PLA and LDPE causing dense CNT aggregation in LDPE. Initial contact of CNTs with PLA reduced aggregation, allowing PLA to infiltrate CNT aggregates during melt-mixing, which influenced the final morphology and electrical conductivity. These findings provide new insights into the fabrication of conductive polymer composites for force sensor applications.

## 1. Introduction

A flexible film with a semi-conductive property is of interest for a force-sensing application. Several composites comprising a conductive filler and a polymer matrix have been investigated [1]. For optimal performance in force-sensing applications, the film must exhibit both flexibility and resilience to repetitive compression forces. Most critically, the film should possess the optimum semi-conductive property that electrically responds when a force is applied. A composite containing a conductive filler at or above its percolation threshold proves optimal for the force-sensing application. This threshold marks the critical concentration at which conductive fillers establish a continuous network within the polymer matrix, resulting in an insulator-to-conductor transition in the composites [2,3,4,5].

Lowering the percolation threshold of a conductive filler/polymer composite is a primary focus for researchers, as it promises to reduce the overall cost of producing conductive components for force sensors. Several strategies could be used to lower the percolation threshold of a conductive polymer composite [1]. Among these, employing nano-conductive fillers with high aspect ratios, such as CNTs, stands out as one of the most promising methods because the percolation threshold of a conductive filler in a polymer matrix is strongly influenced by the filler’s shape. Generally, conductive spherical particles like CB, with an aspect ratio (α) around 1, exhibit a higher percolation threshold compared to high-aspect-ratio materials like carbon fiber and CNT [2]. The formation of a chain-like conducting network of CNTs throughout the polymer matrix [2,6,7] could significantly enhance the electrical conductivity of the composite. However, the relatively high cost of CNTs, despite recent price reductions [2,8], presents a significant ongoing challenge. Consequently, reducing the amount of CNTs required in the production of conductive polymer composites is key to developing cost-effective materials with exceptional electrical conductivity.

Many researchers have observed that conductive fillers incorporated into immiscible polymer blends tend to selectively concentrate within one phase or at the interface of polymer blends [9,10,11]. Introducing electrically conductive fillers to a co-continuous morphology of polymer blend systems could reduce the percolation threshold and effectively improve the electrical properties of the composites due to the dense segregated structure of the conductive filler in either phase [12,13]. This phenomenon is well known as “double percolation” [14]. Liu, Y. et al. [12] demonstrated that incorporating silicon dioxide (SiO_2_) into the poly(lactic acid) (PLA) component effectively enhanced the viscosity and elasticity of the PLA phase. This resulted in a morphological transformation of the polycaprolactone (PCL) phase from dispersed domains to a co-continuous structure with the addition of only 30 wt% PCL. Selective distribution of CNTs in the PCL phase was employed to establish a more effective conductive network, leading to a significant increase in conductivity for the PLA/PCL/CNT composites. The addition of SiO_2_ reduced the percolation threshold of CNT in the PLA/PCL/CNT composite from 0.28 wt% (0.14 vol%) to 0.11 wt% (0.06 vol%), representing a 125% reduction compared to the composite without SiO_2_ incorporation. Pötschke, P. et al. [15] found that a significant reduction in electrical resistivity of CNT-filled polycarbonate/high-density polyethylene (PC/HDPE) blends could be achieved in co-continuous structures with a total CNT loading content of only 0.41 vol%. Al-Saleh, MH. et al. [16] observed that the selective localization of CNTs in the polystyrene (PS) phase in the PC/PS blend, with 0.05 wt% (0.034 vol%) CNT loading content, effectively formed a conductive network within the polymer blend.

Numerous factors, including rheological [17,18,19,20,21], thermodynamic [11,17,20], and kinetic considerations [17,20,21], intricately influenced the final localization of conductive fillers in immiscible polymer blends. The specific interactions between the CNT with each polymer component—associated with the surface energy predicted by the wetting coefficient (ω)—contributed to the thermodynamic distribution of CNTs [22,23,24]. For instance, Chen, J. et al. [11] incorporated the maleic anhydride-grafted ABS (ABS-*g*-MA) into the polycarbonate/acrylonitrile-butadiene-styrene (PC/ABS) blend as a compatibilizing agent. The wetting coefficient calculation revealed a stronger thermodynamic interaction between CNTs and ABS-*g*-MA compared to the interactions between CNT-PC and CNT-ABS pairs. Thus, the thermodynamically favorable localization of CNTs was in the ABS-*g*-MA component, resulting in the final CNTs’ distribution at the interface between the PC and ABS phases. For the PLA/PCL blend system, the PCL phase was thermodynamically more favorable for the CNTs rather than the PLA phase [17,20,25]. With lower melt-viscosity than PLA, the migration of CNTs from the initially existing PLA phase to the PCL component was, therefore, accompanied by the rheological and thermodynamic dominances [17,20]. The kinetics controlling the migration of CNTs from the unfavorable PLA phase toward their favorable PCL phase was time-dependent. Manipulation of melt-mixing sequence and time for the incorporation of the PCL component into the molten PLA/CNTs masterbatch caused the final localization of CNTs in either PLA phase, PCL phase, or interface between PLA and PCL phases [17,20], thereby the conductivity of PLA/PCL/CNT composites fluctuated according to the processing durations [20]. Therefore, careful attention must be paid to the selection of polymer pairs, their viscosities, and the mixing sequence to control filler distribution.

In this study, interest was focused on the immiscible blend of polylactic acid (PLA) and low-density polyethylene (LDPE). LDPE is chosen for its favorable physical properties, including high elongation at break, high impact strength, chemical inertness, low cost, and low density [26,27]. Previous research has shown that the PLA/LDPE blend can form an in situ fiber-reinforced composite at a relatively low PLA loading of 10 wt% [28]. This in situ fiber reinforcement is primarily attributed to the viscosity ratio of the blend being around 1, facilitating favorable in situ fiber formation. Other studies have reported that a co-continuous structure can be formed as the PLA content increases. Djellali, S. et al. [27] found that PLA/LDPE blends with ratios of 60/40 and 40/60 exhibited a dispersed LDPE phase in the continuous phase of PLA, and vice versa, while a 50/50 PLA/LDPE blend had a co-continuous morphology without a phase-in-phase structure. Similar co-continuous blend morphologies have also been observed in 50/50 wt% blends of PLA with linear low-density polyethylene (LLDPE) [29] and high-density polyethylene (HDPE) [30] systems.

To the best of our knowledge, while numerous research papers pay attention to the characterizations and properties of CNT-polymer composites [24,31,32,33,34,35], none have explored the investigation of the percolation threshold of CNT-filled PLA/LDPE blends. Additionally, there has been no examination of the preferential localization of CNTs in co-continuous PLA/LDPE blends influenced by sequential melt-mixing. The ability to control the structure of the PLA/LDPE blends suggests the potential to manipulate the preferential direction for the conductive property of the composite film, which could be valuable for certain applications. The in-depth understanding from the result of this work should prove useful and pave the path for further development of efficient and effective conductive components for force sensor application in the future.

## 2. Materials and Methods

### 2.1. Materials

PLA (Indigo 4043D) is a multi-purpose extrusion grade having a melting point of around 150 °C [28] and was purchased from NatureWorks LLC. (Plymouth, MN, USA). LDPE resin (InnoPlus LD2426H) with a melting temperature (T_m_) of approximately 110 °C [28] was obtained from PTT Global Chemical Public Co., Ltd. (Rayong, Thailand) in a pellet form. Multiwall carbon nanotube (CNT) having a diameter of 12.6 nm and length of 3–12 µm was obtained from Nano Generation Co., Ltd. (Chiang Mai, Thailand). Chloroform and hexane were from RCI Labscan Ltd. (Bangkok, Thailand). Ethanol and glycerol were purchased from DUKSAN (Ansan, Korea), Krungthepchemi Co., Ltd. (Bangkok, Thailand), respectively. Commercial force sensor components were supplied from Shenzhen Lizhuan Technology Co., Ltd., (Guangdong, China).

### 2.2. Sample Preparations

PLA was pre-dried at 70 °C in a hot air oven for 4 h to remove moisture before use. The melt-mixing process of the PLA/LDPE/CNT composites with 50/50 wt% PLA/LDPE were performed in an internal mixer (Haake^™^ Rheomix 3000p, Thermo Fisher Scientific, Frankfurt, Germany) at a temperature of 170 °C for 10 min with a rotation speed of 60 rpm. Table 1 provides an overview of the sequential mixing steps and corresponding sample code names, which include (i) simultaneous melt-mixing of PLA, LDPE, and CNTs for 10 min, (ii) initial melt-mixing of PLA and CNTs for 5 min followed by the addition of LDPE with further melt-mixing for 5 min, and (iii) melt-compounding LDPE and CNTs for 5 min before the introduction of PLA, followed by additional melt-mixing for 5 min. The total melt-mixing time was 10 min for all samples. Afterward, all the samples were hot-pressed into a thin film with a thickness of 200–400 µm at 170 °C under the pressure of 150 psi for 5 min before air-cooling to room temperature. The CNTs with the different loading contents were also incorporated into LDPE during melt-mixing for investigation of the percolation threshold. The CNT loading content of 7.5 wt% was selected to further study the influences of the melt-mixing sequence on the final localization of CNTs and their conductance.

### 2.3. Characterizations

The contact angle measurements with the four different test liquids (i.e., DI water, hexane, glycerol, and ethanal) were determined using the sessile drop technique with a Contact Angle Meter (OCA 20, DataPhysics Instruments GmbH, Filderstadt, Germany) at room temperature (25 °C). The cryo-fractured surface morphology of the composites was analyzed using a scanning electron microscope (SEM, JEOL, JSM6100) with an accelerating voltage of 10 kV. Prior to analysis, all specimens underwent coating with gold. To enhance the clarity of the phase structure of the composites, the PLA phase was etched using chloroform for 30 min at room temperature. The dispersion and preferential localization of CNTs in the composites were visualized using a field-emission scanning electron microscope (FE-SEM, Auriga, Carl Zeiss, Jena, Germany) with an accelerating voltage of 5 kV. Observations were made on the cryo-fractured cross-sectional surfaces of the composites and all specimens were coated with carbon prior to examination. Furthermore, the examination was conducted using a transmission electron microscope (TEM, TALOS F200X, Thermo Scientific, MA, USA) with a 100 kV accelerating voltage. The ultrathin section ranging from 150 to 200 nm was prepared by cutting the cross-sectional specimen by an ultramicrotome and subsequently placed on a copper grid for TEM observation. Atomic force microscopy with conductive tip (AFM, XE-120, Park Systems, Suwon, Korea) was used to visualize the electrically conductive paths of CNTs in the composites with a scanning rate of 0.5 Hz and a direct current of 3 V. The sample was cross-sectionally cut with an ultramicrotome and then connected with the AFM by using a conductive silver paste.

**Figure 1:**
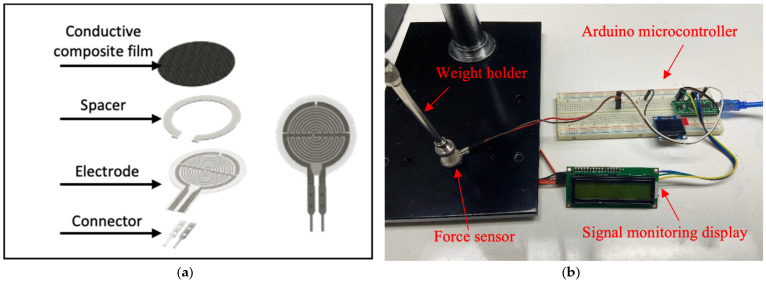
(**a**) The schematic image of commercial force sensor components and (**b**) the instrument setup for sensor signal characterization.

The electrical conductivity of the composites was measured using a 2-point probe instrument (Keithley Instruments Inc., Solon, OH, USA). For these measurements, a commercial force sensor was used, consisting of a conductive top layer and an interdigital electrode bottom layer separated by a spacer, as illustrated in Figure 1a. In the absence of pressure, the spacer maintains separation between the layers, resulting in high resistance. For testing the performance of the PLA/LDPE/CNT composites, we replaced the original conductive layer with our conductive composite specimens. A minimum of five distinct areas on each composite sample film were tested. The sensor signal characterization setup is shown in Figure 1b, employing a voltage divider circuit to measure the sensor’s response signal. The specimens were connected to an Arduino Nano board, which interfaced with an LCD display to present the change in sensing signal in terms of ADC value, voltage, and corresponding sensor resistance. Compression forces ranging from 1 to 100 N were applied using a standard force gauge (SH-500, Digital force gauge, Sundoo, China) mounted on a manual lever-type fixture. By measuring the voltage change, the circuit determined the force applied to the sensor. The force responsiveness of the composites was converted into electrical signals using the Arduino microcontroller. Five specimens were tested and the average values were reported.

## 3. Results and Discussion

### 3.1. Thermodynamic Prediction on the Preferential Localization of CNTs

In a thermodynamic equilibrium state, the specific interactions between CNTs and each component in the composites contribute to the final distribution of CNTs [22,23,24]. Consequently, it is anticipated that the CNTs may be located within either PLA or LDPE or at the interface between the two immiscible polymers. The correlation between surface tension and final distribution of CNTs can be predicted by calculating the wetting coefficient (ω) in the following Equation (1) [14].

(1) $$\omega =\frac{\gamma_{CNT-LDPE\;}-\gamma_{CNT-PLA}}{\gamma_{PLA-LDPE}}$$

Here, $\gamma_{CNT-LDPE}$, $\gamma_{CNT-PLA}$, and $\gamma_{PLA-LDPE}\;$ represent the interfacial surface tension between CNT and LDPE, CNT and PLA, and PLA and LDPE, respectively. When ω is greater than 1, CNTs exhibit a preferential location in the PLA phase. Conversely, if ω is less than –1, CNTs are anticipated to localize within the LDPE component. For cases where ω is between –1 and 1, CNTs are expected to selectively localize at the interface between the PLA and LDPE phases [14,22].

The interfacial surface tension can be calculated from the surface energy values. Two widely employed approaches for this purpose are the harmonic-mean [36] and geometric-mean equations [37], as depicted by Equations (2) and (3), respectively.

(2) $$\gamma_{12}=\gamma_{1}+\gamma_{2}-4\left(\frac{\gamma_{1}^{d}\gamma_{2}^{d}}{\gamma_{1}^{d}+\gamma_{2}^{d}}+\frac{\gamma_{1}^{p}\gamma_{2}^{p}}{\gamma_{1}^{p}+\gamma_{2}^{p}}\right)$$

(3) $$\gamma_{12}=\gamma_{1}+\gamma_{2}-2\left(\sqrt{\gamma_{1}^{d}\gamma_{2}^{d}}+\sqrt{\gamma_{1}^{p}\gamma_{2}^{p}}\right)$$

where $\gamma_{12}$ describes the surface energy between two component interactions. The subscripts 1 and 2 mention to component 1 and 2 (i.e., $\gamma_{PLA-CNT}$, $\gamma_{LDPE-CNT}$, and $\gamma_{PLA-LDPE}$), respectively. The superscript *d* and *p* are the dispersive and polar components of surface energy.

In conjunction with surface energy with contact angle measurement, the OWRK theory has been adopted to calculate the surface energy of each component by separating the interfacial surface tension to disperse nonpolar and polar interactions [36,38]. Thus, the surface energy is the summation of polar and dispersive components [37,39]. Therefore, a minimum of two liquids—with different polarities and known dispersive and polar surface tensions—is required for calculating the surface energy of the PLA and LDPE specimens [39,40]. The OWRK model can be expressed in a linear form (*y* = m*x* + *c*), as shown in Equation (4):

(4) $$\frac{\gamma_{L}\left(1+cos\theta \right)}{2\;\left(\sqrt{\gamma_{L}^{D}}\right)}=\left(\sqrt{\gamma_{s}^{P}}\right)\sqrt{\frac{\gamma_{L}^{P}}{\gamma_{L}^{D}}}+\sqrt{\gamma_{s}^{D}}$$

where $\gamma_{S}$ is the total surface energy and *θ* is the measured contact angle between the liquid and the solid phase. $\gamma_{L}^{D}$ and $\gamma_{L}^{P}\;$ are the liquid’s surface tension of dispersive and polar components. $\gamma_{S}^{D}$ and $\gamma_{S}^{P}\;$ represent the solid’s surface tension of dispersive and polar components. The subscript *L* and *S* relate to the liquid and solid phases, respectively.

**Figure 2:**
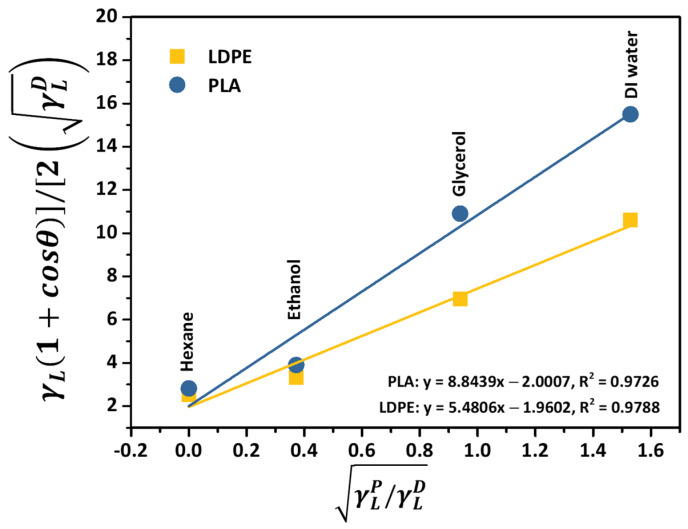
The OWRK plot for the surface energy calculation of PLA and LDPE.

From Equation (4), after plotting the surface tension ($\sqrt{\gamma_{L}^{P}/\gamma_{L}^{D}}$) of the corresponding liquid acquired from the liquid’s surface tension reported in Table 2 versus the cosine term of the measured contact angle of individual solvents ([$\gamma_{L}\left(1+cos\theta \right)]/[2\left(\sqrt{\gamma_{L}^{D}}\right)]$), the slope of the linear function fitted through each data point represents the polar part of the solid’s surface energy, as illustrated in Figure 2. The extrapolation points to the *y*-intercept, using a linear regression line, refers to the dispersive component of the solid’s surface energy. It can be seen that the notable linearity of the plot with an R^2^ value of 0.9726 and 0.9788 indicated a precise determination of the dispersive surface energy of the PLA and LDPE specimens that relied on the OWRK model.

It is important to emphasize that the measurement of the surface free energy of polymers was conducted based on contact angle measurements at ambient temperature. Since interfacial tensions were temperature-dependent, the surface energy of each component was extrapolated with the values at ambient temperature to the melt-mixing temperature of 170 °C according to Equations (5) and (6):

(5) $$-\frac{d\gamma }{dT}=\frac{11}{9}\frac{\gamma_{0}}{T_{c}}\times {\left(1-\frac{T}{T_{c}}\right)}^{\frac{2}{9}}$$

(6) $$\gamma =\gamma_{0}\times {\left(1-\frac{T}{T_{c}}\right)}^{\frac{11}{9}}$$

where $\gamma_{0}$ is the surface tension at 0 K, $T_{c}$ is the critical temperature (K), and T is the temperature of the polymer (K). For most polymers, $T_{c}$ is about 1000 K [23,24].

Table 3 shows the surface free energy for PLA, LDPE, and CNT at the temperature of 25 and 170 °C. The surface free energy of a single CNT was obtained from the literature [42] and it was independent to the temperature [23,24]. The interfacial surface tension among PLA-CNT, LDPE-CNT, and PLA-LDPE was determined utilizing Equations (2) and (3) and wetting coefficient calculated from Equation (1) detailed in Table 4. It was evidenced that PLA contributed a higher polar surface energy (95% polarity)—compared to the LDPE (89% polarity)—due to the presence of ester groups in the main chain as well as the hydroxyl alcohol and carboxylic acid groups at the chain ends of PLA. The surface energy of LDPE and CNT ($\gamma_{LDPE-CNT}$) was closed together as compared to PLA and CNT ($\gamma_{PLA-CNT}$), meaning that the wetting ability of CNT with LDPE was higher than that of CNT and PLA. The wetting coefficients calculated from both the harmonic and geometric means were lower than –1. This prediction concluded that the CNTs were a thermodynamically preferable localization in the LDPE phase.

### 3.2. Preferential Localization of CNTs in PLA/LDPE/CNT Composites

**Figure 3:**
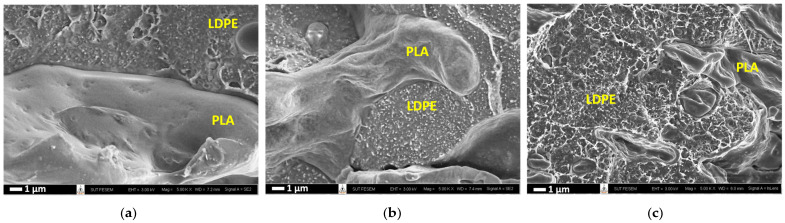
FE-SEM micrographs of cryo-fractured surface of PLA/LDPE/CNT composites with CNT loading of (**a**) 5 wt%, (**b**) 7 wt%, and (**c**) 7.5 wt% (5 k magnification).

**Figure 4:**
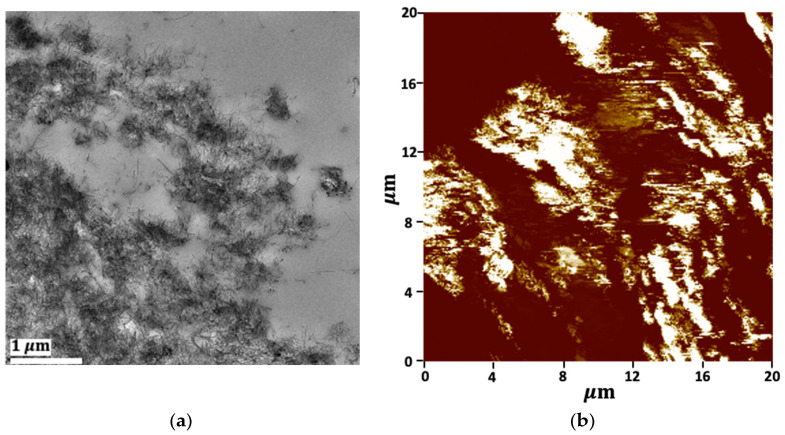
The morphology of PLA/LDPE/CNT composites with 7.5 wt% CNT observed by (**a**) TEM (6.8 k magnification) and (**b**) conductive AFM (+3 V).

As would be evaluated later, the electrical percolation threshold of PLA/LDPE/CNT composites was about 5.56 wt% CNT addition. The CNT loading contents of 5, 7, and 7.5 wt% were deliberately chosen to reveal the selective localization of the PLA/LDPE/CNT composites, as prepared by the simultaneous melt-mixing procedure. In Figure 3, the PLA phase exhibited a clear cryo-fractured surface (the smooth area). The CNTs as white dispersed particles were observed to mainly localize and randomly distribute throughout the LDPE phase. The denser white spots were observed when the amount of CNTs increased from 5 to 7.5 wt%. The formation of CNT networks throughout the LDPE phase was clearly observed in the TEM image, as shown in Figure 4a. Additionally, the electrically conductive paths of the CNTs localized in the LDPE phase were shown in a conductive AFM image, displayed as the bright areas in Figure 4b. This evidence confirmed that CNTs were selectively localized within the LDPE phase rather than in the PLA phase.

### 3.3. Electrical Conductivity and Percolation Threshold

The construction of a double percolation structure through a co-continuous immiscible polymer blend aimed to attain high electrical conductivity with minimizing the conductive filler loading [9,10,12,14,15,16]. By taking the aforementioned percolation threshold into account, it is directly related to an amount of conductive filler incorporated in the polymer matrix in which the electrical conductivity of a composite significantly increases and insulator–conductor transition occurs [2,3,4]. As shown in the previous section, CNTs have a stronger affinity with the LDPE phase. Therefore, LDPE/CNT and PLA/LDPE/CNT composites prepared by a simultaneous melt-mixing procedure were selected to investigate the conductivity threshold with various CNT loading contents.

**Figure 5:**
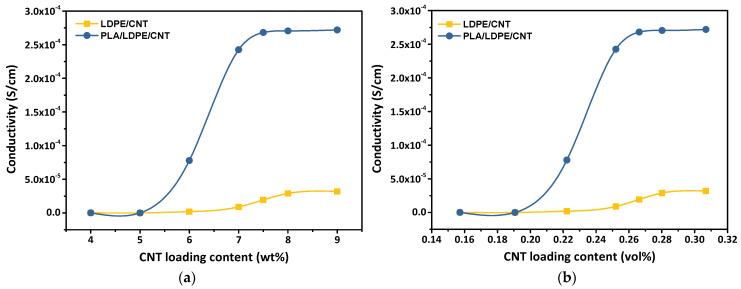
Electrical conductivity of the CNT/LDPE and CNT/PLA/LDPE composites with different CNT loading contents expressed by (**a**) weight fraction (wt%) and (**b**) volume fraction (vol%).

Figure 5a,b show the electrical conductivity of LDPE/CNT and PLA/LDPE/CNT composites as a function of weight fraction and volume fraction of CNT loading contents, respectively. The volume fraction of CNT loading content ($V_{CNT}$) can be calculated from Equation (7) below:

(7) $$V_{CNT}={\left(\frac{\rho_{r}}{wt_{CNT}}-\rho_{r}+1\right)}^{-1}$$

where $\rho_{r}$ is the ratio between the densities of the CNT and the polymers ($\rho_{CNT}/\rho_{p}$) [2]. In the current case, the density of PLA, LDPE, and CNT was 1.24, 0.94, and 0.21 g/cm^3^, respectively.

It was observed that the CNT/LDPE composite showed an insulating behavior at the CNT loading contents lower than 7 wt% (0.25 vol%), resulting from lacking an electrically conductive network of CNTs. The electrical conductivity of the LDPE/CNT composite slightly increased as the CNT loading contents went beyond 7 wt% due to the forming of interconnected CNT clusters. On further increasing CNT loading contents, the contact resistance between fillers limited the maximum conductivity of the composites [2]. Thus, the electrical conductivity leveled off since the CNT loading content of 8 wt% (0.28 vol%), showed an almost constant conductivity of 2.89 × 10^−3^ S/cm. On the other hand, the localization of CNTs in the LDPE phase of a co-continuous PLA/LDPE system not only reduced the S-shaped curve corresponding to cement percolation of CNTs but also significantly improved the electrical conductivity of the composites. The electrical conductivity of PLA/LDPE/CNT composites increased early since the CNT loading content of 5 wt% (0.21 vol%) turned into the plateau region after 7.5 wt% CNT addition (0.29 vol%).

According to a power law fit derived from the classical percolation theory [2], the relationship between the electrical conductivity (σ) and $V_{CNT}$ of the composite was examined by Equation (8):

(8) $$\sigma =\sigma_{0}{\left(V_{CNT}-V_{c}\right)}^{t}$$

where $V_{c}$ is the volume fraction (vol%) of CNT filler at the percolation threshold and $\sigma_{0}$ is a constant that is typically assigned to the plateau conductivity of the fully loaded composite and *t* is a critical exponent depending on the geometry of the network which can be taken from the slope of the plot of logσ versus log($V_{CNT}-V_{C}$) [43].

**Figure 6:**
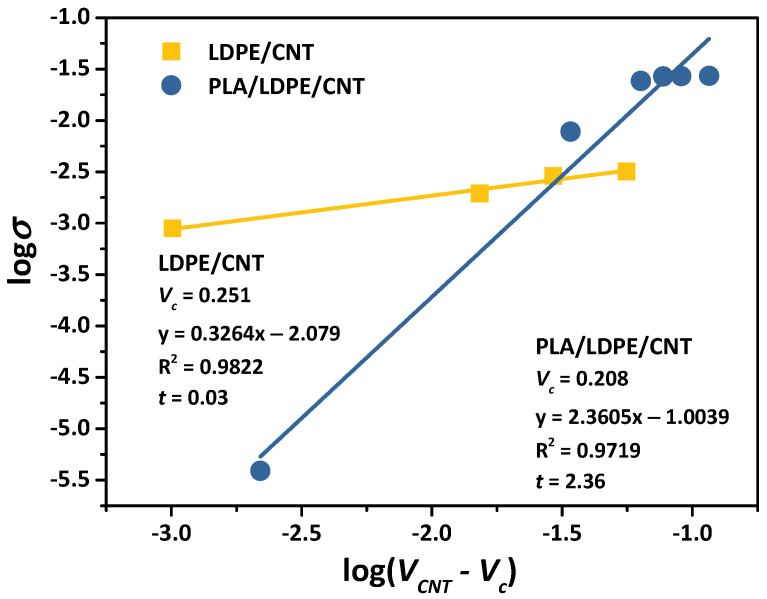
A plot of logσ versus log($V_{CNT}-V_{C}$) of LDPE/CNT and PLA/LDPE/CNT composites. The *V_c_* was varied until the best linear fit and the critical exponent (*t*) were reported.

To determine the percolation threshold of the composites, the experimental $V_{C}$ resulted in an S-shaped curve region and was varied until the best correlation factor (R^2^) for the plot of logσ versus log($V_{CNT}-V_{C}$) [2,5,44,45]. Fitting results are shown in Figure 6. The best fitting to the experimental values showed a percolation threshold of 0.251 vol% (6.95 wt%) for the LDPE/CNT composite. This finding was similarly observed in McNally, T. et al.’s report [46] in which the percolation threshold PE/CNT composites prepared by melt-blending was approximately 7.5 wt% CNT incorporation. Additionally, it was reported that the PLA/CNT composite prepared by the direct melt-blending process exhibited a percolation threshold of 0.46 vol% [47]. On the other hand, PLA/LDPE/CNT composites exhibited a lower percolation threshold of 0.208 vol% (5.56 wt%), marking an approximately 80% reduction of CNT loading content compared to the LDPE/CNT composite, and a 55.65% reduction compared to the PLA/CNT composite reported in the literature [47].

In an insulating matrix, spherical particles exhibit a critical exponent (*t* value) in the range 1.1–1.3 corresponding to a 2D system and between 1.6 and 2.0 for a 3D system [13,43]. In contrast, CNT-polymer composites usually have a critical exponent exceeding 2 due to the high aspect ratio of fiber shape compared to spheres [44,45]. It has been well documented that the critical exponent of the CNT-polymer composites ranging from 1.3 to 4 and was predominant at around 2 [5]. The critical exponent identified in this study was 2.36 for the PLA/LDPE/CNT composites, suggesting the 3D establishment of a CNT conductive network in the LDPE phase. Conversely, the LDPE/CNT composite, with a critical exponent of 0.33, theoretically indicated a 1D percolating network of CNTs [48]. This critical exponent was notably lower than many values presented in the literature [5], although some authors have reported similar observations [48]. In experimental composite systems, the presence of weakly connected branches within the filler networks was identified as a factor contributing to the reduction in the critical exponent. Zhang, R. et al. [49], for instance, noted a decline in critical exponents from 1.98 to 1.6 when CNT/polyurethane-urea composites were stretched from zero strain to around 5% strain. This change was attributed to the alteration of the dimensionality of the conductive network from 3D toward 2D.

In heterogeneous blend systems, many mechanisms were used to explain the electrical conductivity of the CNT-polymer composites such as (i) Hopping/tunneling mechanism: conduction occurred through electron hopping between CNTs without the necessity for physical contact. Conductive paths are established within the composites due to the quantum tunneling effects between neighboring CNTs by a tunneling effect. The inter-CNT distance should be sufficiently close to enable electron transfer through the interphase surrounding CNTs (tunneling spaces)—a thin layer of insulating polymer—toward the adjacent CNTs; (ii) Percolation: the formation of CNT connective networks expands through the entire system, creating microscale conductive networks that facilitate the electrical conductivity paths of CNT-polymer composites. [50,51,52,53]. Owing to the connection of the LDPE phase in the co-continuous morphology and the percolation of the CNTs, a double percolation in the LDPE phase significantly enhanced the electrical conductivity of the PLA/LDPE/CNT composite (2.68 × 10^−2^ S/cm). This improvement was 13.8 times higher than the electrical conductivity of the LDPE/CNT composite (1.94 × 10^−3^ S/cm) at the same CNT loading content of 7.5 wt% (Figure 5). In the case of the LDPE/CNT composite, the strong affinity between LDPE and CNTs, coupled with the high viscosity of LDPE, led to the agglomeration of CNTs within the LDPE matrix (further explained by the rheological factor, Section 3.5). This resulted in conductive paths being separated by a thick polymer barrier, as evidenced by the low value of the critical exponent. The hopping/tunneling of charges faced considerable difficulty under these conditions. Consequently, the LDPE/CNT composite exhibited relatively low conductivity, even at high CNT loading contents. It could be reasonably anticipated that at much higher CNT loading contents, a second threshold might be reached when the CNT inclusions come into direct contact, thereby eliminating contact resistance.

### 3.4. Mixing Sequences

In the construction of polymer composites, the fillers are randomly distributed within the polymer matrix, resulting in a relatively high percolation threshold [54]. Researchers have explored diverse processing to lower the percolation threshold of the composites [55,56]. Utilizing CNT-filled co-continuous polymer blends offers advantages compared to a single-phase polymer composite, particularly through the phenomenon of double percolation. In co-continuous polymer blends, if CNTs are situated in one phase, only a minimal number of CNTs are required to establish the conductive network. Some researchers have also identified the mixing sequence as a crucial parameter for controlling particle localization in polymer blends [17,57,58]. In this case, the CNTs were first incorporated into either PLA or LDPE before subsequently blended with another. The 0.75 wt% CNT loading content was selected to study the influences of the melt-mixing sequence on the CNTs’ localization due to the highest electrical conductivity achieved at the lowest maximum CNT loading content (Figure 5a).

**Figure 7:**
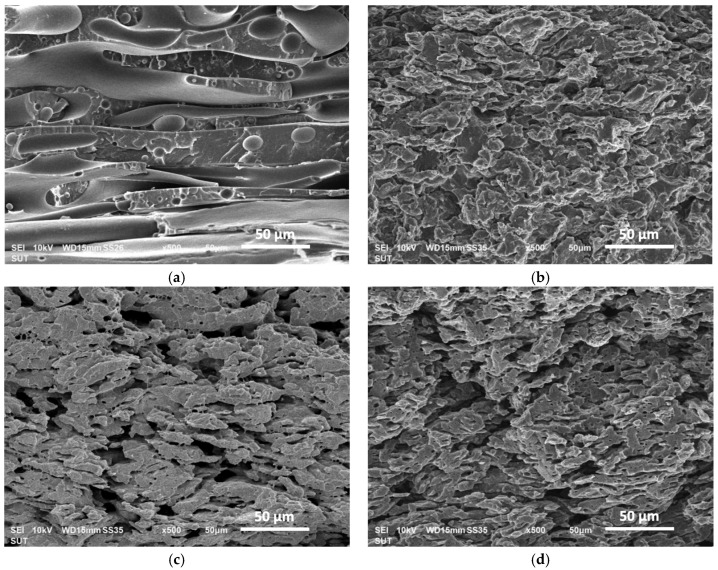
SEM micrograph of the PLA-etched surface morphology of (**a**) PLA/LDPE blend, (**b**) PLA/LDPE/CNT, (**c**) [PLA + CNT]/LDPE, and (**d**) PLA/[LDPE + CNT] composites (0.5 k magnification).

Figure 7 illustrates the PLA-etched surface morphology of the PLA/LDPE blend and its composite with a 7.5 wt% CNT addition. The PLA/LDPE blend exhibited a co-continuous morphology, while the incorporation of CNTs resulted in a refined co-continuous microstructure and the phase thickness for both phases decreased. Moreover, the different melt-mixing sequences showed insignificant differences of the composite morphology as compared to those produced by simultaneous melt-mixing. This refined co-continuous microstructure of the composites could be attributed to the compatibilizing effect from CNTs—reducing the surface tension of the two polymers [59]. A similar observation regarding morphological changes with CNT addition was reported in various CNT-polymer composite systems [59,60,61]. These findings suggested that CNTs effectively facilitated the interpenetration of PLA and LDPE phases and suppressed the coalescence of both phases during melt-mixing.

**Figure 8:**
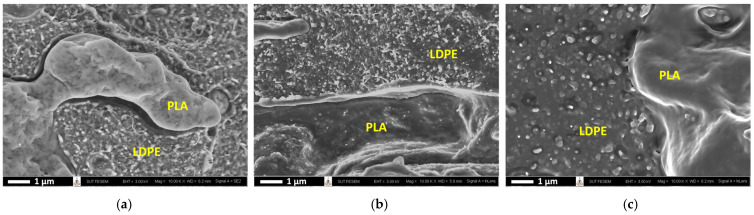
FE-SEM images of a cryo-fractured surface of (**a**) PLA/[LDPE + CNT], (**b**) [PLA + CNT]/LDPE, and (**c**) PLA/LDPE/CNT composites with 7.5 wt% CNT addition (1 k magnification).

The final localization of the CNTs in the composites prepared with different melt-mixing sequences observed by FE-SEM is shown in Figure 8. In the two-step melt-mixing process, the PLA/[LDPE + CNT] composite—where CNTs were firstly contracted with the LDPE phase—explored that the final localization of CNTs was predominant in the LDPE phase due to thermodynamic preference. However, most of the CNTs in the [PLA + CNT]/LDPE composite—where CNTs initially dispersed in the PLA phase—resided in the LDPE component and some amount of CNTs was observed in the PLA component. In a very familiar system to our case [62], due to carbon black (CB) having a high affinity with the HDPE component, the deportation of CBs from the PLA/CB masterbatch to the HDPE phase progressively increased with increasing the melt-mixing time. Additionally, the CNTs in the PLA/HDPE/CNT composites were reported to be selectively localized in the HDPE domain [63]. Therefore, in this context, the migration of CNTs from the PLA phase, which exhibited a lower affinity toward the preferred LDPE phase, was incomplete due to the limited melt-mixing time (5 min remaining) for the predominant transfer of the CNTs to the LDPE phase. Consequently, a portion of the CNTs persisted in the PLA component. In contrast, the PLA/LDPE/CNT composite prepared by simultaneous melt-mixing demonstrated a distribution of CNTs mostly within the LDPE phase. This occurrence was because a sufficient melt-mixing time (10 min) facilitated the mobilization of CNTs to primarily concentrate in the LDPE phase. Thus, the CNTs in the PLA phase were barely observed in the PLA/LDPE/CNT composite, as compared to that of the [PLA + CNT]/LDPE composite.

### 3.5. Discussion on the Competition between Thermodynamic and Kinetic Factors on the Final Localization of CNTs

The introduction of a filler into the binary polymer blend systems can result in three potential spatial distribution scenarios. First, the filler may be randomly dispersed within the two phases of the blend. Second, it might predominantly localize within either phase of the two polymer phases. Third, it can be concentrated at the face-interphase of the two polymer blends [57]. The final localization of fillers is influenced by various factors such as (i) thermodynamic factor: associated with the surface energy predicted by the wetting coefficient (ω); (ii) kinetic factor: linked to the melting temperature of each component and processing condition; (iii) rheological factor: related to the viscosity of polymer matrix during processing; (iv) geometry factor: related to geometry of the nanoparticles; and (v) chemical factor: owing to the functionalization for further or in situ chemical reaction between the filler and polymer [17,19,57]. In this case, the CNTs used in this work were used as received without any prior chemical modification. Consequently, the chemical factor was disregarded, and emphasis was placed on the consideration of thermodynamic, kinetic, rheological, and geometric factors.

**Figure 9:**
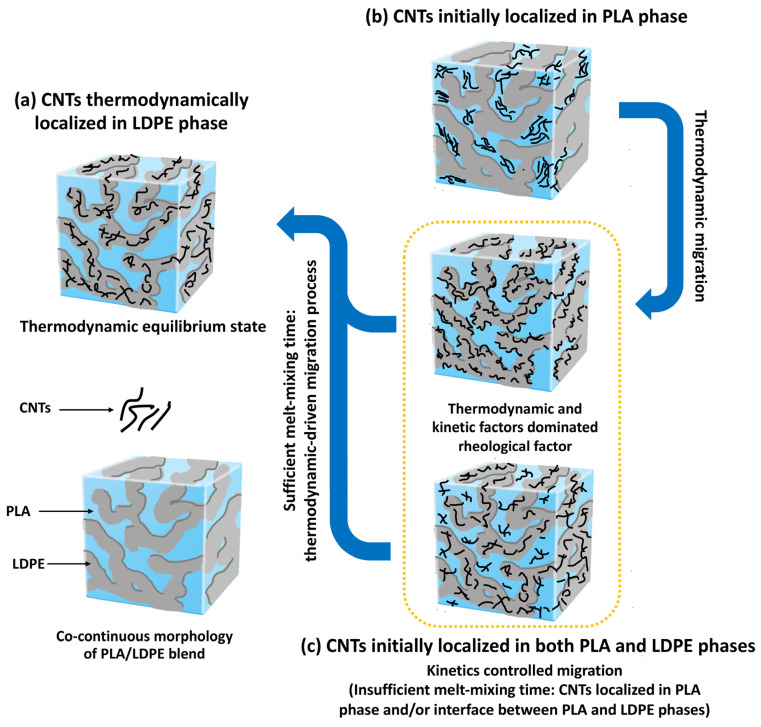
Illustrations of possible localization of CNTs predicted by dominated factors: (**a**) CNTs thermodynamically localized in LDPE phase, (**b**) CNTs initially localized in PLA phase, and (**c**) CNTs initially localized in both PLA and LDPE phases.

Figure 9 is the schematic that illustrates the possible localizations of CNTs in the co-continuous morphology of the PLA/LDPE blend system. Theoretical predictions derived from thermodynamic considerations suggested the preferential localization of CNTs in the LDPE component with good agreement with the results from morphological analysis. The preferential location of CNTs was governed by CNT-LDPE affinity through the interfacial energy, independently of the mixing sequence. Therefore, selective localization of CNTs in the PLA/[LDPE + CNT] composite was addressed concerning the thermodynamic dominant, as depicted in Figure 9a. Regarding the melt-mixing sequence, in the first moment of simultaneous melt-mixing, the CNTs were early “wet” with the LDPE phase due to the lower T_m_ of around 110 °C. The higher T_m_ of PLA of about 150 °C resulted in a solid state of PLA. Before the complete melting of both phases, some CNTs migrated to the LDPE phase due to thermodynamic government and some localized at the interface boundary of both components. As further continuous melt-mixing, most of the CNTs transferred to the LDPE phase and thus the similar morphology of PLA/LDPE/CNT and PLA/[LDPE + CNT] composites was observed. Many studies indicated that CNTs tended to localize in the lower melt-viscosity phase, attributing to the hindrance effect (discussed in Section 3.6) [17,18,19,20]. However, PLA showed lower melt-viscosity than that of LDPE at the processing temperature [64,65]. Such selective localization of CNTs in our case was addressed concerning the thermodynamics and kinetics effects that dominated the rheological property.

On the other hand, in the [PLA + CNT]/LDPE composite, when the CNTs were initially localized in the less favorable PLA component, the competition between thermodynamics and kinetics factors existed. The PLA chains absorbed onto the CNT surface during the first melt-mixing step with PLA. Upon the subsequent addition of LDPE (5 min later), the adsorbed PLA chains were replaced by LDPE chains through the migration mechanism of CNTs driven by thermodynamics, as shown in Figure 9b. However, the complete migration of CNTs across the interface of both components required a sufficient melt-mixing time for achieving the wetting-induced transfer process [19]. Huang, J. et al. [20] monitored the deportation of CNTs from the less favorable PLA component to the more favorable polycaprolactone (PCL) phase using TEM. The localization of CNTs at the interface was found after melt-mixing with PCL for 4 min and took over 10 min to complete deportation. A similar observation was shown in the report of Aguiar, J. et al. [17]. The CA migrated from the PLA phase to the phase-interface between PLA and HDPE components after incorporation of the HDPE in the molten PLA/CA composite for 3 min. The CA entered the HDPE phase and formed an effective conductive network—leading to stable conductivity—as the mixing time reached 10 min. As compared to our blend system, CNTs with a higher aspect ratio could penetrate the interface more rapidly and efficiently compared to the lower aspect ratio of CA during melt-mixing, a phenomenon known as “Slim-Fast Mechanism” [66]. However, the presence of CNTs in the polymer matrix predominantly existed as agglomerates or coil-like shapes, potentially resulting in a low aspect ratio [19,66]. Consequently, the short processing period of 5 min was insufficient for CNTs to complete migration to the LDPE phase, resulting in some CNTs remaining in the PLA component. Therefore, the kinetic effects related to the melt-mixing sequence and time overcame the thermodynamic and rheological migration of CNTs in the case of the [PLA + CNT]/LDPE composite, as represented in Figure 9c. For a longer melt-mixing time, the total CNTs could transfer to the LDPE phase and the thermodynamic equilibrium state was achieved.

### 3.6. Discussion on the Competition between Thermodynamic and Rheological Effects on the Final Localization of CNTs

As aforementioned, the rheological factor normally dominated among other factors and the CNTs showed a tendency to migrate to the lowest viscosity phase. This phenomenon was attributed to the hindrance effect, resulting from the increased melt-viscosity of the blend system by the formation of entanglements between CNTs-CNTs and CNT-polymers. Consequently, polymer chains with lower viscosity were relatively easier to diffuse and penetrate into CNT-aggregated domains compared to those in a high-viscosity matrix [19]. Such in the case of CNTs-filled polyethylene/polyethylene oxide (PE/PEO) blends [23], although the PE phase was energetically more favored than the PEO phase—thermodynamic prediction CNTs localized in the PE phase. The CNTs selectively localized in the PEO phase due to the lower melt-viscosity, as compared to the PE. Li, Q. et al. [67] revealed that the localization of CB was mostly in the thermodynamically less-favored PP phase rather than in the ultra-high molecular weight poly(ethylene) (UHMWPE), due to the very high melt-viscosity of the energetically favored UHMWPE phase making it difficult for CB particles’ migration.

In our case, during melt-mixing, PLA and LDPE underwent fusion and exhibited characteristics of immiscible fluids, whereas the CNTs remained in a solid state throughout the complete melting of the polymers. In this state, the CNTs were wet through the liquid–solid interface with the polymer melt. The motivation for the thermodynamic migration of CNTs lay in the favorable interaction between CNTs and LDPE much more than between CNTs and PLA. Although PLA has lower melt-viscosity to that of LDPE, the migration of CNTs to the higher melt-viscosity LDPE component was evidenced. Therefore, the CNTs localized in the LDPE phase were predominantly driven by thermodynamic dominance rather than rheological factor.

**Figure 10:**
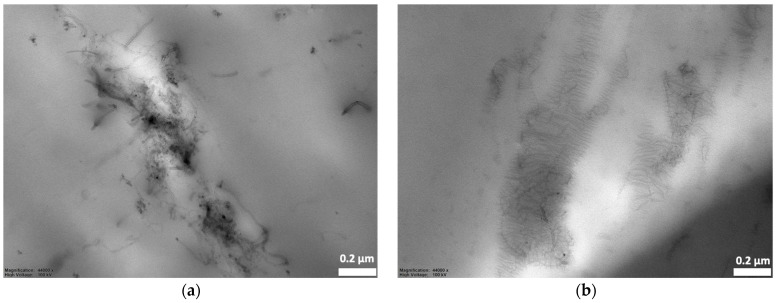
TEM micrographs of a cryo-fractured surface of (**a**) PLA/[LDPE + CNT] and (**b**) [PLA + CNT]/LDPE composites with 7.5 wt% CNT addition (44 k magnification).

However, the rheological factor also showed an influence on the morphological difference of the resulting CNTs’ alignment. The outcomes were observed in the TEM observation, as shown in Figure 10. The final localization of the CNTs in the PLA/[LDPE/CNT] composite primarily existed in coiled configurations or were connected to other coiled tubes, forming micro-agglomerations throughout the LDPE phase occupation. Whereas the [PLA + CNT]/LDPE composite exhibited a thin layer of polymer between CNTs. The CNT agglomerates could be penetrated by the unfavorable PLA component during initial melt-mixing. LDPE—with a relatively strong affinity to CNTs—adsorbed on the CNT surface upon concurrent desorption from PLA during additional melt-mixing. As a result, the CNTs were separated and encapsulated by LDPE, thereby affecting the morphological and electrical conductivity differences.

### 3.7. Force Sensor Application

**Figure 11:**
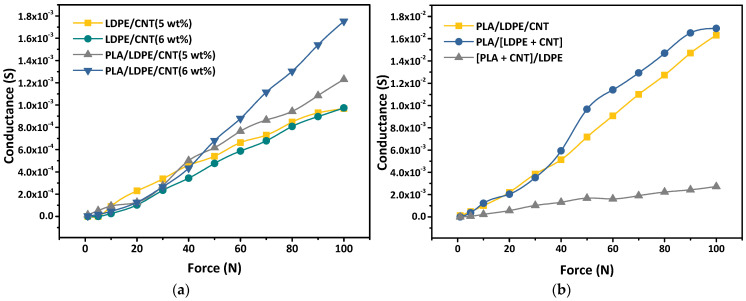
The influences of (**a**) CNT loading contents and (**b**) melt-mixing sequences on the electrical responsiveness property of the composites.

Figure 11a displays the relationships between conductance and applied force of LDPE/CNT and PLA/LDPE/CNT composites with the CNT loading contents of 5–6 wt%, ranging between lower and higher percolation threshold of PLA/LDPE/CNT composites (5.56 wt%). In LDPE/CNT composites, the conductance exhibited nearly linear growth with increasing applied forces and remained independent of the CNT loading contents. Despite the CNT loading being below the percolation threshold (6.95 wt% for LDPE/CNT composite), the application of applied force led to the formation of conductive paths. This was attributed to the reduced inter-CNT distance facilitating electron transfer. A similar trend was observed in the PLA/LDPE/CNT composite with a 5 wt% CNT loading approaching its percolation threshold, but its conductance surpassed that of LDPE/CNT composites. Consequently, a greater number of conductive paths could be formed resulting in higher conductance. In contrast, the PLA/LDPE/CNT composite with a 6 wt% CNT loading content beyond its percolation threshold showed the highest conductance among them. This could be attributed to the electrical responses under the applied force that were related to the electrical conductivity of the PLA/LDPE/CNT composites. However, the conductance of all samples had less sensitivity when the applied forces were lower than 20 N.

Figure 11b displays the influence of the melt-mixing sequences on the force sensor of the PLA/LDPE/CNT composites. In this case, the CNT loading content of 7.5 wt% was beyond the percolation threshold. Connection of CNTs enabled the electronic transport between CNT networks. As expected, the PLA/[LDPE + CNT] composite showed the highest conductance under the same applied force. This was ascribed by the effective network formation of CNT conductive paths related to the double percolation mechanism. However, although the PLA/LDPE/CNT composite had a similar CNT distribution in the LDPE phase to those of the PLA/[LDPE + CNT] composite, the conductance of the PLA/LDPE/CNT composite was lower. A possible explanation was that the CNTs were initially localized in both the LDPE phase and phase-interface of the partially molten PLA phase and molten LDPE phase during the early simulations melt-mixing step, as described previously. Some CNT agglomerates could be separated by the PLA component due to lower viscosity. During the adsorption–desorption mechanism, some amounts of the single CNT were encapsulated by the LDPE phase leading to the potential barrier to the inter-CNT hopping/tunneling processes. Thus, the CNT conductive networks were imperfect and lower in degree. Therefore, the conductance of the PLA/LDPE/CNT composite was lower than the PLA/[LDPE + CNT] composite. However, CNTs in the PLA/LDPE/CNT composite had enough time for deportation from the PLA to the LDPE phase more than the [PLA + CNT]/LDPE composite. It was evident that the [PLA + CNT]/LDPE composite exhibited the lowest conductance. The electrical conductivity of the CNT-polymer composites was reported to be strongly related to the CNT loading contents due to denser CNT networks [68]. The incomplete migration of CNTs from the PLA to LDPE phase affected the lower content of CNT localized in LDPE, thereby affecting the conductance.

Furthermore, it was found that the conductance of the PLA/[LDPE + CNT] composite became stable at high applied forces of 90–100 N. It was believed here that increasing the applied forces no longer significantly enhanced the composite’s electrical conductivity because the composite had reached a state where most possible conductive paths were already established. As a result, further conductance changes had minimal effect and led to the saturation of the electrical signal. However, both PLA/LDPE/CNT and PLA/[LDPE + CNT] composites exhibited a large force-sensitive range, indicating that the fabricated composite films in this work hold great promise for force-sensing applications.

From our point of view, the 3D printing process showed a good candidate method in producing the pressure/force sensor made of CNT composites [69,70,71]. The resulting sensors demonstrated excellent linearity under strain and were highly sensitive to various tactile parameters. The sensitivity and detection range of the sensors were influenced by the printing speed and the amount of carbon fillers used. However, a significant drawback of 3D printing technology is that it produces rough surfaces due to the layer-by-layer deposition process. This results in lower surface quality and inferior mechanical and thermal properties, especially in relation to the printing direction [70]. Therefore, a flexible film with a semi-conductive property fabrication from compression molding or blown film extrusion is of interest for a force-sensing application. Our results demonstrated that the composites had almost linearly electrical responsiveness with force. Constructing a double percolation threshold of CNT-filled PLA/LDPE composites reduces the amount of CNT loading contents in the system. However, the literature reported that controlling CNTs’ localization at the interface between co-continuous polymer components not only exhibits the ultra-low percolation thresholds, but also shows high conductivity value [11,20]. In future studies, monitoring CNTs’ migration from the PLA phase to the LDPE phase to evaluate the optimal blending time for positioning CNTs at the interface of co-continuous PLA/LDPE blend will be investigated as well as the performance and durability of the composite sensor.

## 4. Conclusions

In this work, CNTs were incorporated into a co-continuous PLA/LDPE blend for constructing a conductive polymer composite for force-sensing application. Predictions based on thermodynamic considerations strongly indicated the favored localization of CNTs in the LDPE phase. The co-continuous morphology of the immiscible PLA/LDPE blend played an important role in the double percolation phenomenon. When the conductive fillers are selectively localized in the LDPE phase, another phase plays the role of volume exclusion, leading to an increase in the concentration of the CNTs in the LDPE component. This finding can be confirmed by the presence of CNTs in the PLA phase by the SEM, FE-SEM, TEM, and AFM observations. CNT-filled co-continuous polymer blends provided advantages compared with single-phase polymer composites through the double percolation phenomenon. Therefore, the percolation threshold significantly decreased from 0.251 vol% (6.95 wt%) for the LDPE/CNT composite to 0.208 vol% (5.56 wt%) for the PLA/LDPE/CNT composite with a high electrical conductivity reaching 2.68 × 10^−2^ S/cm. Depending on the melt-mixing sequence, a competition between thermodynamic and kinetic factors could be observed when CNTs initially encountered the thermodynamically less-favorable PLA phase. The rheological factor was dominated by both thermodynamic and kinetic effects. Therefore, the different electrical properties of the composites with different melt-mixing sequences were observed, thereby affecting the electrical responsiveness of the sensor.

## Figures and Tables

**Table 1:** Sample code names and the melt-mixing sequences.

Nomenclature	Components on 1st Mixing Step	Components on 2nd Mixing Step
PLA/LDPE/CNT	PLA, LDPE, and CNT	-
PLA/[LDPE + CNT]	LDPE and CNT; [LDPE + CNT]	Added PLA after 5 min melt-mixing
[PLA + CNT]/LDPE	PLA and CNT; [PLA + CNT]	Added LDPE after 5 min melt-mixing
LDPE/CNT	LDPE and CNT	-

**Table 2:** The surface energy of the solvents used for the wettability test [41].

Solvent	Surface Tension (mJ/m^2^)		
	$\mathrm{Dispersive}\;(\gamma_{L}^{D})$	$\mathrm{Polar}\;(\gamma_{L}^{P})$	$\mathrm{Total}\;(\gamma_{L}$)
DI water	21.8	51.0	72.8
Hexane	18.4	0	18.4
Glycerol	34.0	30.0	64.0
Ethanol	18.8	2.6	21.4

**Table 3:** The surface free energy of PLA, LDPE, and CNT.

Components	$\mathrm{Dispersive}\;\mathrm{Surface}\;\mathrm{Energy}\;(\gamma_{S}^{D})$ (mJ/m^2^)		$\mathrm{Polar}\;\mathrm{Surface}\;\mathrm{Energy}\;(\gamma_{S}^{P})$ (mJ/m^2^)		$\mathrm{Total}\;\mathrm{Surface}\;\mathrm{Energy}\;(\gamma_{S})$ (mJ/m^2^)		Polarity (%)
	25 °C	170 °C	25 °C	170 °C	25 °C	170 °C	
PLA	4.0	1.57	78.2	30.62	82.2	32.19	95
LDPE	3.8	1.50	30.0	11.76	33.9	13.26	89
CNT [42]	17.6		10.2		27.8		37

**Table 4:** Interfacial surface tension ($\gamma_{12}$) and wetting coefficient (*ω*) evaluated at 170 °C.

Parameters	Harmonic-Mean Equation	Geometric-Mean Equation
$\gamma_{PLA-CNT}$ (mJ/m^2^)	23.629	14.139
$\gamma_{LDPE-CNT}$ (mJ/m^2^)	13.671	8.869
$\gamma_{PLA-LDPE}$ (mJ/m^2^)	8.396	4.430
Wetting coefficient (*ω*)	–1.186	–1.190
Theoretical prediction of CNTs’ localization	LDPE phase	LDPE phase

## Data Availability

The original contributions presented in the study are included in the article; further inquiries can be directed to the corresponding author.
